# Multimodal analysis of the differential effects of cyclic strain on collagen isoform composition, fibril architecture and biomechanics of tissue engineered tendon

**DOI:** 10.1177/20417314221130486

**Published:** 2022-10-31

**Authors:** Adam J Janvier, Emily G Pendleton, Luke J Mortensen, Daniel C Green, James R Henstock, Elizabeth G Canty-Laird

**Affiliations:** 1 Department of Musculoskeletal and Ageing Science, Institute of Life Course and Medical Sciences, University of Liverpool, Liverpool, UK; 2 Department of Animal and Dairy Science, University of Georgia, Athens, GA, USA; 3 The Medical Research Council Versus Arthritis Centre for Integrated Research into Musculoskeletal Ageing (CIMA), Liverpool, UK

**Keywords:** Tendon, collagen, cyclic strain, collagen isoforms, collagen fibril

## Abstract

Tendon is predominantly composed of aligned type I collagen, but additional isoforms are known to influence fibril architecture and maturation, which contribute to the tendon’s overall biomechanical performance. The role of the less well-studied collagen isoforms on fibrillogenesis in tissue engineered tendons is currently unknown, and correlating their relative abundance with biomechanical changes in response to cyclic strain is a promising method for characterising optimised bioengineered tendon grafts. In this study, human mesenchymal stem cells (MSCs) were cultured in a fibrin scaffold with 3%, 5% or 10% cyclic strain at 0.5 Hz for 3 weeks, and a comprehensive multimodal analysis comprising qPCR, western blotting, histology, mechanical testing, fluorescent probe CLSM, TEM and label-free second-harmonic imaging was performed. Molecular data indicated complex transcriptional and translational regulation of collagen isoforms I, II, III, V XI, XII and XIV in response to cyclic strain. Isoforms (XII and XIV) associated with embryonic tenogenesis were deposited in the formation of neo-tendons from hMSCs, suggesting that these engineered tendons form through some recapitulation of a developmental pathway. Tendons cultured with 3% strain had the smallest median fibril diameter but highest resistance to stress, whilst at 10% strain tendons had the highest median fibril diameter and the highest rate of stress relaxation. Second harmonic generation exposed distinct structural arrangements of collagen fibres in each strain group. Fluorescent probe images correlated increasing cyclic strain with increased fibril alignment from 40% (static strain) to 61.5% alignment (10% cyclic strain). These results indicate that cyclic strain rates stimulate differential cell responses via complex regulation of collagen isoforms which influence the structural organisation of developing fibril architectures.

## Introduction

Tendons play a key role in the musculoskeletal system acting as force transducers between muscle and bone. Mechanical stimulation is essential in the development and maintenance of tendon structure and function, and resident tenocytes are highly mechanosensitive.^1^ The primary mechanical force experienced by native tendon is tensile stimulation, driven by forces generated by muscular contraction.^2^ During development human tendon is thought to experience small amounts of cyclic strain, coupled with constant strain resulting in an increase of up to 25% of the initial length,^3^ whilst mature tendons are exposed to cyclic strain of up to 6%.^2^ The absence of tensile stimulation during in vivo embryonic growth of chicks has been shown to impair the development of the entire joint including tendons, highlighting the importance of mechanical stimulation for tissue generation.^4^

During embryonic development the modulus and ultimate tensile strength of tendons increases,^5,6^ a trend that continues postnatally until tendons are fully mature.^7–9^ Such properties are independent of increases in tendon length and diameter during tendon growth, which also improve extrinsic tensile biomechanical properties. The extracellular matrix of healthy tendon is composed primarily of collagen, which comprises 60%–85% of the dry weight.^10^ Approximately 95% of the collagenous protein is type I collagen,^11^ which forms string-like fibrils primarily aligned with the long axis of the tendon, and which provide tendons with mechanical durability and strength.^12^ During embryonic development fibril diameters increase from around 30 to 80 nm^13^ but have a predominantly unimodal diameter distribution. Mature tendons display a bimodal or trimodal fibril diameter distribution with fibril diameters reaching approximately 10 times that of embryonic tendon.^14–16^ Tendons comprise a hierarchical structure with fibrils grouping together to form fibres, and fibres together with cells making up the tendon fascicles, themselves joined by an interfascicular extracellular matrix.

Minor collagens of tendon comprise fibrillar types III, V, XI and FACIT (fibril-associated with interrupted triple-helix) collagens XII, and XIV.^11^ Types V and XI form heterotypic (mixed) fibrils with type I collagen in tendon.^17^ Type III reduces heterotypic fibril diameter and is more abundant in elastic and distensible tissues.^18^ During injury and ageing the ratio of type III to type I increases, thereby altering tendon mechanical properties.^19^ Types V and XI nucleate fibril assembly,^20^ with collagen type V influencing the diameter and number of fibrils assembled.^21^ Types XII and XIV are located at the surface of collagen fibrils with type XIV^22^ primarily present in developing tendon and replaced by collagen type XII during tendon maturation and ageing.^23^ The role of these collagen isoforms on de novo fibrillogenesis in bioengineered tendons is currently unknown, and correlating their relative abundance with functional measures is a promising method for characterising optimised bioengineered tendon grafts.

Mechanical stimulation has been applied in tendon bioengineering using an array of different scaffolds, bioreactors and conditions.^24,25^ Tendon- and ligament-like tissues can be recreated using tendon or ligament fibroblasts in fibrin gel scaffolds, which are replaced with a cell-generated, linear collagenous extracellular matrix.^1–4,26–29^ Human mesenchymal stem/stromal cells (MSCs) are commercially available and have the potential to recreate human tendon-like tissue, with future applications in the development of autologous bioengineered tendon replacements. MSCs grown in fibrin hydrogels also produce a collagenous tissue resembling embryonic tendon.^30^ Such encapsulated MSCs increase expression of COL1A1 and the tenocyte marker THBS4,^31,32^ and lose the ability to respond to osteogenic induction.^30^

Whilst collagen hydrogels are often used for tendon tissue engineering, reconstituted fibrils have irregular diameters are not uniformly orientated, and cultures can contain mixtures of cell-synthesised and reconstituted fibrils.^33^ Fibrin hydrogels have been shown to be superior to collagen hydrogels for tenogenesis, with improved tenogenic gene expression, ECM alignment, packing density of fibrils and increased linear modulus when cultured with 2.4% strain for 14 days.^29^ Implantation of scaffold-free self-assembled tendon constructs has been shown to increase tensile strength and collagen content.^34^ We therefore hypothesised that an appropriately biomimetic rate of intermittent, cyclic uniaxial tensile strain could improve the biomechanical properties of cell-engineered tendon-like tissue, whilst maintaining a tendon-like fibril alignment and collagen expression profile. The wide range of strain rates, frequency and duration of tensile stimulation for bioengineered tendon in published studies has previously been reviewed by ourselves and others.^25^

In the present study cell-engineered tendon-like tissues were generated using human mesenchymal stem cells (hMSCs) in fibrin hydrogels, which contracted around 3D printed attachment frames, replacing fibrin with collagen to yield tendon-like tissues after 14 days. To apply cyclic strain, 3D printed bioreactor chambers^25^ connected to an Ebers-TC3 bioreactor were employed. The tendon-like tissues within their frames were then incorporated into the bioreactor for 21 days, with one chamber for each condition (control, 3%, 5% and 10%) and cyclic strain delivered at 0.5 Hz for 5 h per day. These conditions were chosen based on a detailed evaluation of the existing literature to identify commonality in approach, and relevance to a biomimetic strain rate for an immature tendon.^25^ A comprehensive multimodal end point analysis was performed using histology, qPCR and Western blotting for collagen isoform expression, transmission electron microscopy to quantify collagen fibril diameter and analysis of collagen fibre alignment with a fluorescent collagen probe (CNA35) and second harmonic generation imaging. Tensile testing was then used to measure viscoelastic and failure properties of the cell-engineered tendon-like tissues in response to biomimetic cyclic strain.

Our objectives in this study were to conduct a multi-modal analysis of tissue engineered tendon with sufficient experimental power to resolve the impacts of the complete collagen isoform composition on fibrillogenesis and relate this to functional outcomes, that is the emergent mechanical properties of the de novo tissues. Our hypothesis was that the generation of tendon-like tissues by hMSCs occurs by a partial recapitulation of developmental processes in which an array of supplementary and transitory isoforms support the development of primarily type-I fibrils. The fidelity of this recapitulation may be important in the overall biomechanical properties of the tissue, and therefore its suitability for use in clinical applications. We also aimed to compare an array of quantitative imaging techniques for examining collagen architectures at different scales, and assess the merits of non-destructive (label-free) imaging techniques such as Second Harmonic Imaging as a tool for monitoring maturation in engineered tissues.

## Results

### 5% cyclic strain alters expression of collagen types III, XI and XII and tenogenic markers

**Figure 1.**
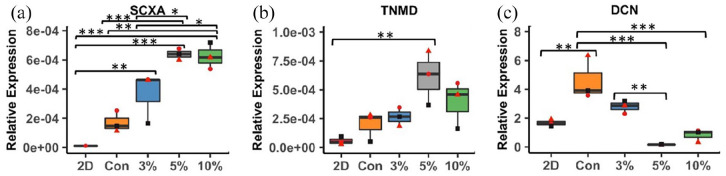
Gene expression analysis of tendon differentiation genes and proteoglycan ECM gene. (a) Scleraxis, (b) Tendomodulin, and (c) Decorin. *R*^2^ = 0.9629 and 0.9808 (*p* < 0.01) between control, 3% and 5% strained groups and control, 3% and 10% strain groups for SCXA respectively. Values shown normalised to YWHAZ. *n* = 3 experimental repeats from three separate experimental runs (*n* = 2 technical repeats). Significance measured using one-way ANOVA with Tukey’s post hoc test and multiple comparisons (*p < 0.05, ** *p* < 0.01, *** *p* < 0.001).

**Figure 2.**
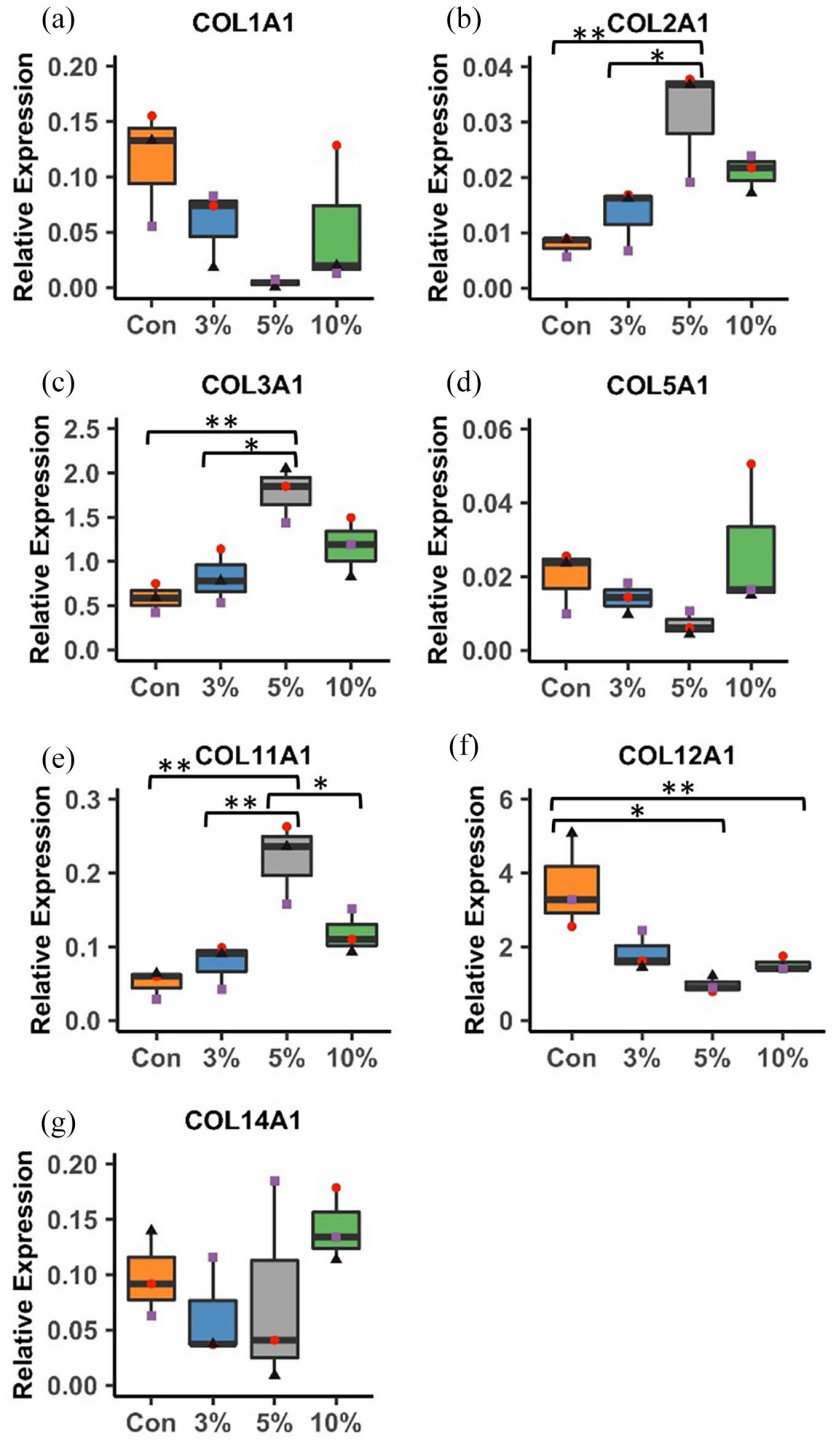
Gene expression analysis of collagen isoforms in cell-engineered tendon-like tissue. a) COL1A1, b) COL2A1, c) COL3A1, d) COL5A1, e) COL11A1, f) COL12A1, g) COL14A1. Relative expression is normalised to YWHAZ housekeeper. Box plots show median and interquartile range. *n* = 3 experimental repeats, three points per group (red, purple and black) designate three separate experimental runs. Significance was measured using one-way ANOVA with Tukey’s post hoc test and multiple comparisons (*p < 0.05, ** *p* < 0.01).

hMSCs were characterised (Supplemental Figure S1) and used to generate cell-engineered tendon-like tissues. After the 2 week contraction and 3 week loading phases, qPCR was carried out for tenogenic differentiation markers (Figure 1), a panel of tendon-specific collagen isoforms and a non-tenogenic marker COL2A1 (Figure 2). Scleraxis (SCXA) was more highly expressed with 3%, 5% or 10% strain than in monolayer (*p* = 0.006, *p* < 0.001, *p* < 0.001 respectively) and with 5% and 10% strain than with stasis (*p* < 0.001 and *p* = 0.001) or 3% strain (*p* = 0.033 and *p* = 0.044) with only one sample from the monolayer expressing detectable SCXA (Figure 1(a)). Tenomodulin (TNMD) was more highly expressed with 5% cyclic strain than in monolayer culture (*p* = 0.009) (Figure 1(b)). Expression of decorin, a key proteoglycan present in tendon, was higher in the control group than in monolayer (*p* = 0.005) or with 5% (*p* < 0.001) or 10% (*p* < 0.001) strain (Figure 1(c)). Expression of COL2A1 (Figure 2(b)), COL3A1 (Figure 2(c)) and COL11A1 (Figure 2(e)) was significantly higher with 5% cyclic strain than 3% cyclic strain (COL2A1; *p* = 0.033, COL3A1; *p* = 0.015, COL11A1; *p* = 0.006) or than in static conditions (COL2A1; *p* = 0.008, COL3A1; *p* = 0.004, COL11A1; *p* = 0.002). COL11A1 expression was also higher with 5% than 10% cyclic strain (*p* = 0.036). Conversely, COL12A1 expression was significantly lower with 5% and 10% strain than in the static control (*p* = 0.008 and *p* = 0.029 respectively) (Figure 2(f)). COL1A1 and COL5A1 followed a similar pattern to COL12A1 though differences were not statistically significant (Figure 2(a) and (f)).

**Figure 3.**
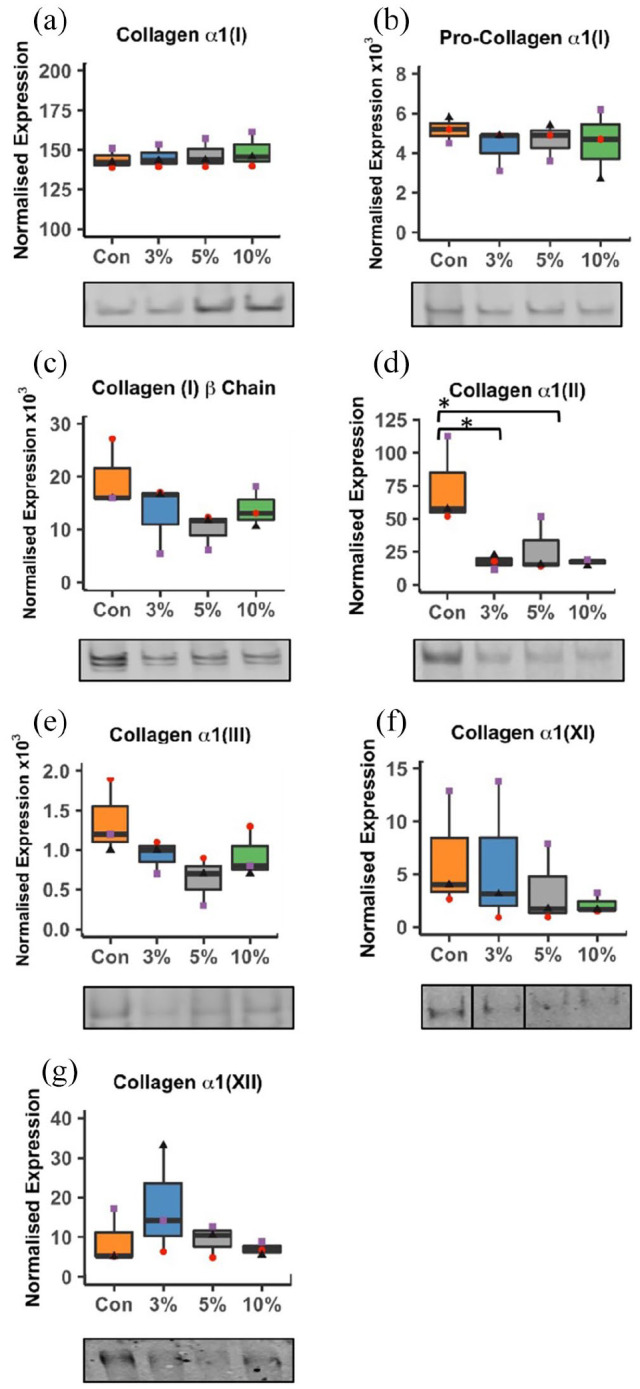
Western blot analysis of tendon collagen isoforms in cell-engineered tendon-like tissue. a) Type I collagen α1 chain, b) type I procollagen α1 chain, c) type I collagen β chain, d) type II collagen α1 chain e) type III collagen α1 chain, f) type XI collagen α1 chain, g) type XII collagen α1 chain. Proteins were quantified using densitometry, with representative images of Western blots show below each graph. Black bars indicate that a membrane has been cropped for the representative image. *R*^2^ = 0.9953 for collagen α1(I) (*p* < 0.01), *R*^2^ = 0.9906 for collagen α1(XI) (*p* < 0.01), and *R*^2^ = 0.8138 for collagen α1(XII) (*p* < 0.01) between the 3%, 5%, and 10% groups. Values shown are normalised to total protein. Box plots show median and interquartile range. *n* = 3 repeats, three points per group (red, purple and black) designate three separate experimental runs. Significance was measured using one-way ANOVA with Tukey’s post hoc test and multiple comparisons, collagen α1(I) was measured with Kruskal-Wallis test (*p < 0.05).

Protein levels of collagen isoforms in the sample were measured using western blot and quantified by densitometry (Figure 3). The reported tendon collagen repertoire (types I, III, V, XI, XII, XIV) was investigated, however types V and type XIV were not detected in any samples. Collagen type II was again considered as a negative marker. Procollagen α1(I) and the cross-linked β1(I) form were both detected on the western blot membrane for collagen Iα1 and were included in the analysis of protein expression. No significant differences in type I, III, XI or XII collagen protein abundance were detected between conditions (Figure 3(a)–(c) and (e)–(g)). Type II collagen was significantly less abundant in the 3% and 10% strain groups as compared to the static control (*p* = 0.035 for both) (Figure 3(d)).

### 10%. cyclic strain increases collagen fibre alignment and sample mineralisation

**Figure 4.**
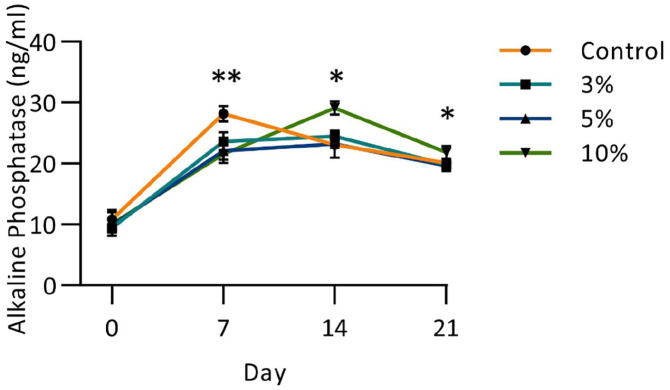
Alkaline phosphatase expression of cell-engineered tendon-like tissue during cyclic loading phase. Day 0 marks the end of the contraction phases and sees the lowest ALP expression with no difference between loading condition. At day 7 the control group had a significantly higher ALP expression than all other groups (*p* < 0.01). The 10% strain group had significantly higher ALP expression than all other groups at both day 14 (*p* < 0.01) and day 21 (*p* < 0.05). *n* = 6. Significance measured using two-way ANOVA with repeat measures and Tukey’s post hoc test (*p < 0.05, ** *p* < 0.01, *** *p* < 0.001).

Qualitative histological analysis of hMSCs cultured under cyclic tensile loading indicated greater cell alignment with increasing strain but no apparent difference in Alcian blue staining between loading regimens (Supplemental Figure S2). Mineralisation was detected in the control group and in the 10% strain group by Alizarin Red staining. Alkaline phosphatase activity of the cell culture media was assayed at 7-day time points during the loading phase. Peaks in activity were detected at day 7 for the control (*p* = 0.0012 vs 3% and *p* < 0.001 vs both 5% and 10% strain) and for the 10% strain group at day 14 (*p* = 0.0017, *p* < 0.001 and *p* = 0.0107 vs control, 3% and 5% strain respectively) and day 21 (*p* = 0.0407, *p* = 0.0305 and *p* = 0.0168 vs control, 3% and 5% strain respectively) (Figure 4).

**Figure 5.**
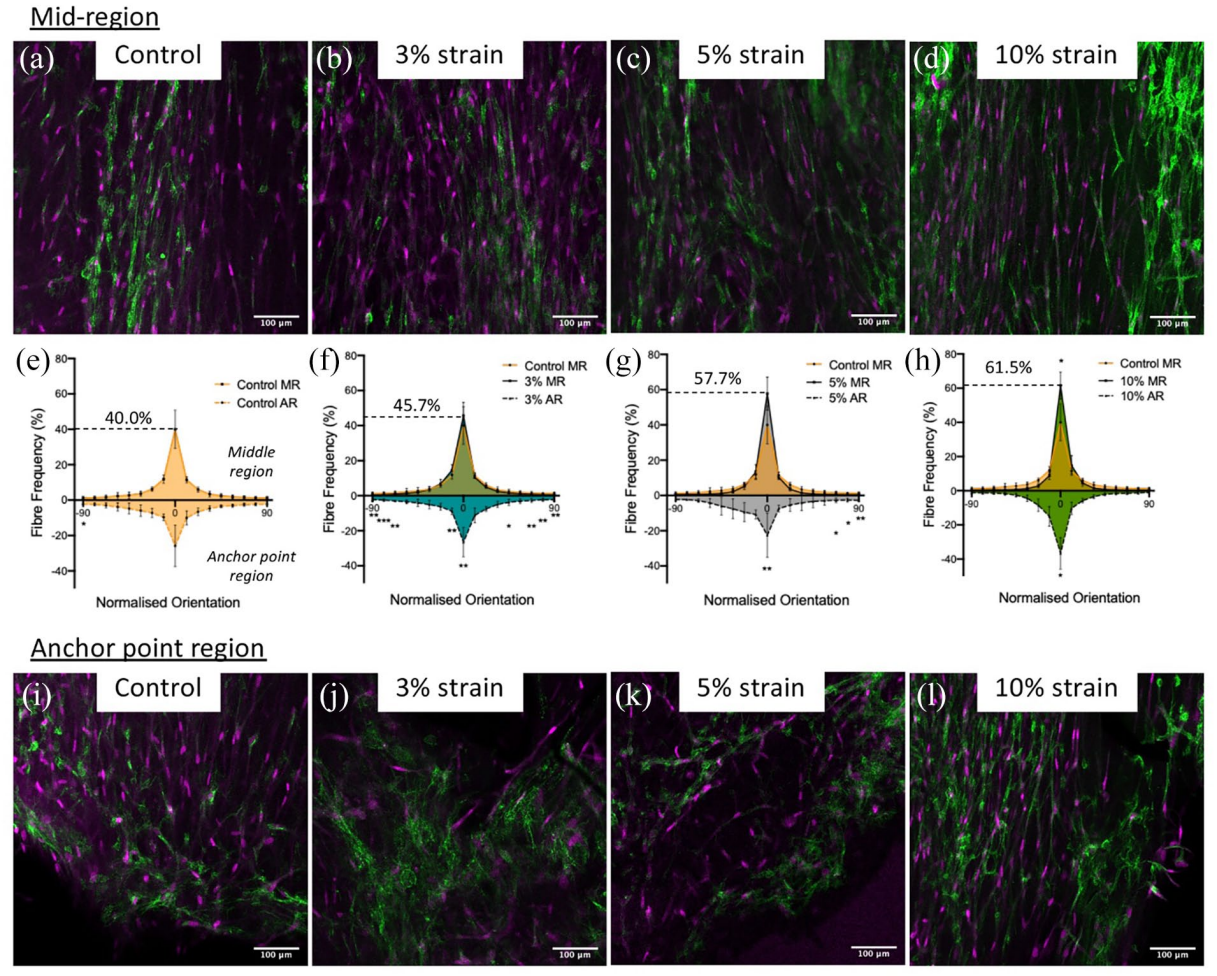
Confocal images of cell-engineered tendon-like tissue showing collagen fibril alignment. Collagen was visualised using CNA35 fluorescent probe (green) and cell nuclei counterstained with DAPI (shown as magenta) in the anchor point (AR) region (a–d) and the middle (MR) region (I-L) of tendon-like tissues cultured without dynamic strain ((a and i); control) or with 3% cyclic strain (b and j), 5% cyclic strain (c and k) or 10% cyclic strain (d and l). The ImageJ plug-in (OrientationJ) was used to visualise and quantify fibril orientation parallel to the strain axis (0° = parallel, shown as vertical in these images) in the both regions. Graphs (e–h) show the frequency distribution of fibres aligned with the strain axis, with the mid-region (MR) shown above the *x*-axis and the anchor point region (AR) shown below the *x*-axis. An overlay of the control group mid-region (in orange) is shown in (f–h) for comparison. Images are representative of *n* = 3 experimental repeats and scale bars in (a–d and i–l) represent 100 µm. Error bars in (e–h) represent standard deviation. Statistical significance cyclic strain versus static (control) denoted above the *x*-axis in (e–h) and mid-point versus anchor point denoted below the *x*-axis was determined using one-way ANOVA with multiple comparison analysis and Tukey’s post hoc test (*p < 0.05, **p < 0.01, ***p < 0.001).

Collagen alignment was measured in CNA35 collagen-probe stained samples from all four stimulation groups (Figure 5). Representative images are shown for the control (a and i), 3% strain (b andj), 5% strain (c and k) and 10% strain (d and l) groups in the mid-region (MR) (a–d) and anchor point region (AR) (i–l). Fibre orientation was measured after normalising to 0° and the percentage frequency distribution of the orientation shown for the control (e), 3% strain (f), 5% strain (g) and 10% strain (h) groups, with the control superimposed in (f–h). Representative images of the visualised orientations are show in Supplemental Figure S3. The control mid region versus the cyclically strained mid regions are shown above the x-axis whilst the anchor point region for the cyclically strained groups are shown below the x-axis. For each cyclically strained condition there was significantly increased alignment in the mid region compared to anchor point region at 0° (*p* = 0.008, 0.002 and 0.019 for 3%, 5% and 10% strain respectively) (below x-axis, Figure 5(e)–(h)). All the strained groups had a more aligned mid region compared to the control mid region, however only the 10% strain group was significantly more aligned (*p* = 0.016) (above x-axis, Figure 5(h)), with 61.5% as compared to 40% alignment in the control group. Alignment in the 3% and 5% strain groups was intermediate between the control and 10% group at 45.7%, and 57.7% respectively.

**Figure 6.**
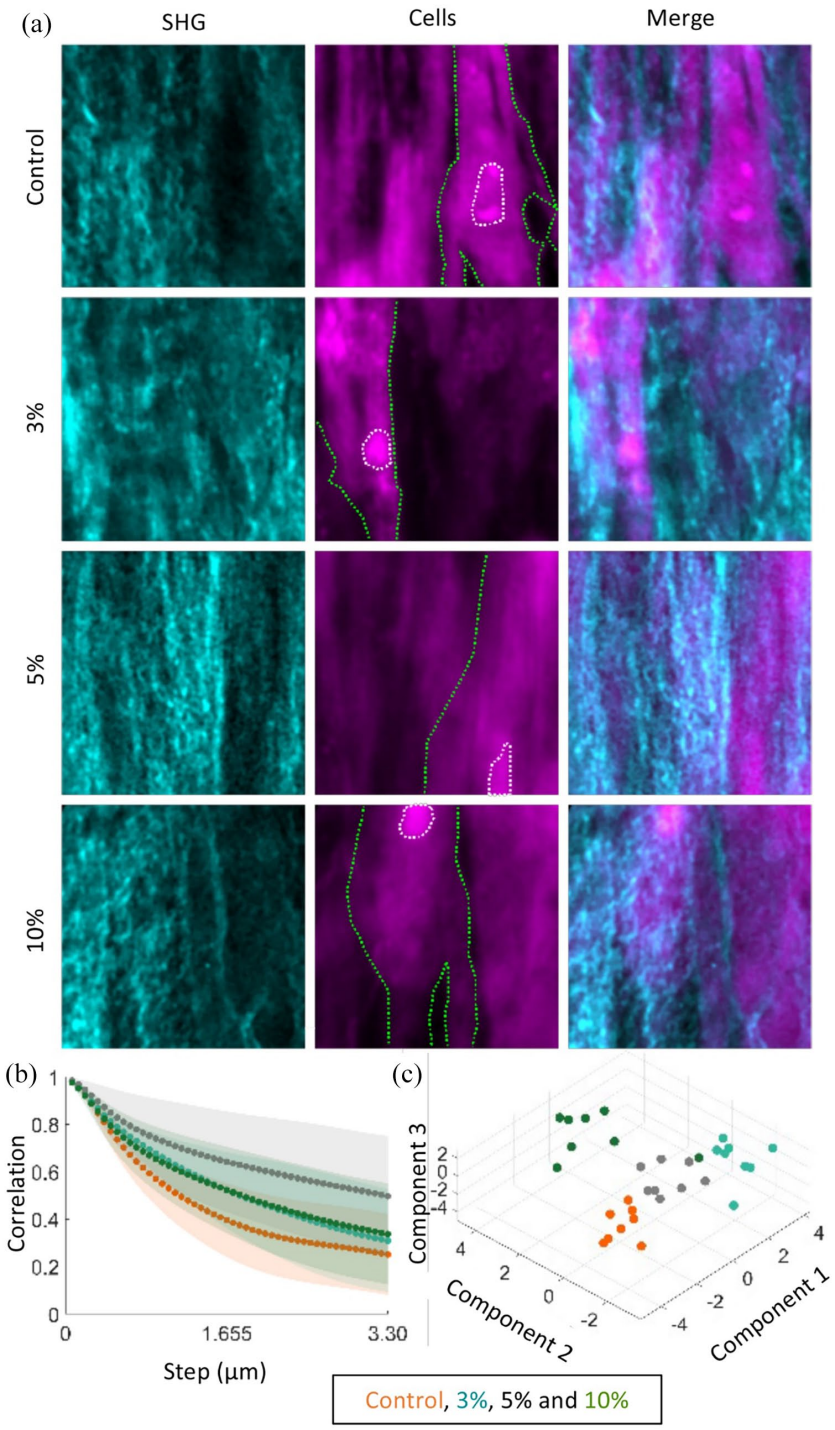
GLCM analysis of SHG images describe differences among strain groups. (a) SHG detected collagen (cyan) deposition from cells (magenta; green outline: cell body, white outline: nucleus). The textural analysis of the collagen structures was be captured with GLCM. (b) Correlation, an example GLCM parameter, shows difference in response among the four groups when analysed along the dominant axis of collagen orientation. Dots are the mean while the shaded area is the standard deviation. The GLCM data was analysed with PLS-DA (c). The strain groups are separated into strain and no strain groups along the first component and then separated by the amount of strain along the second component.

Samples were imaged by two-photon microscopy (Second Harmonic Generation, SHG) to determine the effect of loading on collagen organisation (Figure 6(a)). Without cyclic strain, the collagen SHG signal seems to predominantly assemble in a single direction, but with waves visible within the fibres. The addition of cyclic strain during culture increases the directionality of collagen fibres, with the edges of collagen fibres produced at 3% strain not as well defined as the edges in 5% and 10% strain. In all instances, the long axis of the collagen fibres seems to lay parallel to the long axis of the cells. All images were then analysed using the Grey Level Co-occurrence Matrix (GLCM) measures contrast, correlation, energy and homogeneity at 0°, 45°, 90° and 135° to the pixel of interest at distances up to 3.3 µm away. Here, we show an example of the GLCM parameter correlation response across images and stress groups (Figure 6(b)). With the addition of cyclic strain, the correlation measure curves retain higher values even microns away from the pixel of interest. We then analysed all GLCM measures using a partial least squares discriminant analysis (PLS-DA, Figure 6(c)). Along component 1, the samples are separated into strain and no strain groups. The addition of components 2 and 3 allow for the separation of the strain groups into clusters by the quantity of strain they received. Our model has a misclassification rate of 6.25% using the leave-one-out cross-validation method. Thus, these data support that collagen organisation increases with the addition of mechanical stress, and GLCM texture analysis is able to effectively classify SHG collagen images based on the level of mechanical stress in the samples.

### 3%. cyclic strain reduces collagen fibril diameter but increases tensile strength

**Figure 7.**
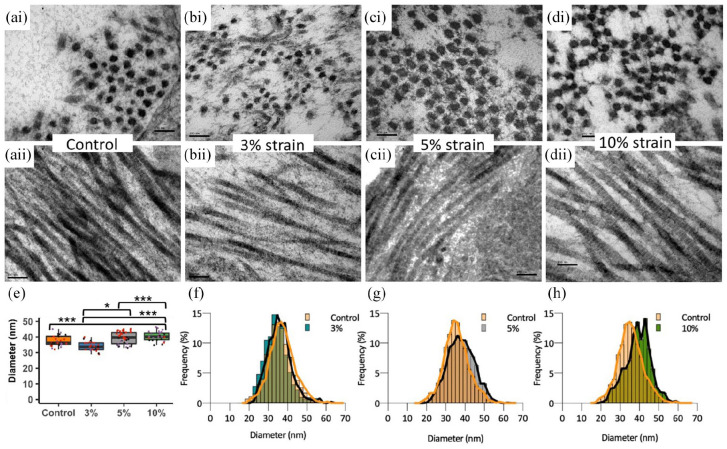
TEM images of cell-engineered tendon-like tissues showing collagen fibril diameter. Upper images (ai–di) show transverse cross-sections of tendon-like tissue, lower images (aii–dii) show longitudinal sections of tendon-like tissue, all after 21 days of intermittent cyclic tensile strain. Analysis of individual fibres in transverse sections by ImageJ (e) showed differences in median fibre diameter with the extent of tensile strain. Data was generated from *n* = 3 experimental repeats of each condition by analysing multiple images from each sample. Data from the same samples are indicated by identical coloured markers within each strain group. Fibril diameters are shown as frequency distributions for (f) 3% strain, (g) 5% strain and (h) 10% strain, with the control group (orange) overlaid for comparison. Scale bars in (a–d) represent 100 nm. Statistical significance was determined using a linear mixed model (*p < 0.05, ***p < 0.001).

Transmission electron microscopy was used to determine the collagen fibril diameter using images of transverse sections of cell-engineered tendon-like tissues (Figure 7(ai)–(di)). Longitudinal sections indicated fibrils had the anticipated surface banding pattern (Figure 7(aii)–(dii)). Fibrils in the 3% strain group had a narrower median diameter of 33.8 nm as compared to 36.3 nm for the control (Figure 7(e) and (f)). The median fibril diameter in the 5% strain group was 39.7 nm (Figure 7(e) and (g)), significantly greater than that of the 3% strain group (*p* = 0.0456). 10% strain increased median fibril diameter as compared to all groups (*p* < 0.001) to 40.2 nm (Figure 7(e) and (h)).

**Figure 8.**
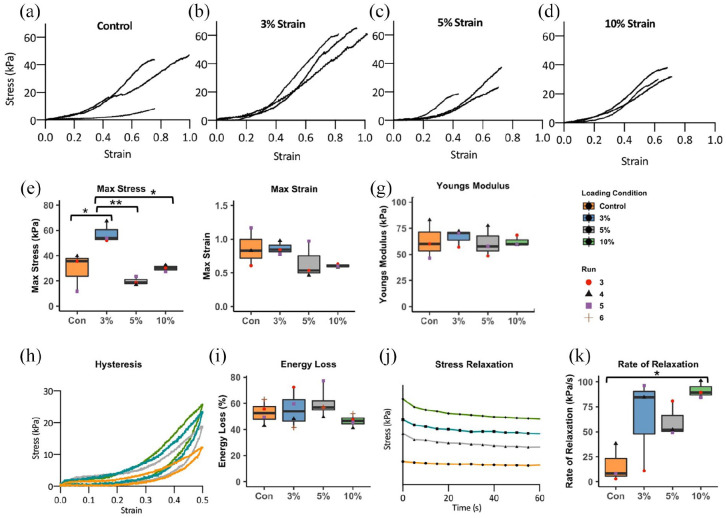
Mechanical testing of cell-engineered tendon-like tissue samples. The upper row of graphs (a–d) shows the stress versus strain profiles for all cell-engineered tendon-like tissue samples after 21 days of intermittent cyclic tensile strain. The maximum stress (e), maximum strain (f) and Young’s modulus (g) were all calculated using the stress versus strain profiles (a–d). The 3% strain group showed the highest maximum stress. The maximum strain and Young’s modulus displayed no significant change across groups. The hysteresis response (h) shows the recorded strain during sample loading and unloading. The percentage energy loss (i) depicts how much energy is lost between the loading and unloading phase for which there were no significant differences between groups. Representative stress relaxation profiles (j) (conditions offset for clarity) showed that all groups displayed this viscoelastic characteristic whilst the rate of stress relaxation (k) was highest in the 10% strain group (*p* < 0.05 vs control). Error bars represent standard deviation of the mean from the experimental replicates (*n* = 3 except for hysteresis where *n* = 4). Statistical significance was determined using one-way ANOVA with multiple comparisons and Tukey’s post hoc test (*p < 0.05, **p < 0.01).

To determine how cyclic strain influences biomechanical function, the tensile and viscoelastic properties of the engineered tendon-like tissues were measured. No significant differences in construct diameters were identified between experimental groups (*p* > 0.1). The average diameter for all samples was found to be 2.43 ± 0.32 mm. In testing to failure (Figure 8(a)–(d)) bioengineered tendon-like tissues grown under 3% cyclical strain showed a significant increase in maximum stress, compared to the control (Figure 8(e)). The 3% strain group had the highest mean maximum stress (57.7 kPa) being twofold higher than the control (*p* = 0.018) and 10% (*p* = 0.022) groups and 2.9-fold higher than that of the 5% group (20 kPa) (*p* = 0.004). Whilst there were no significant differences in mean maximum strain or elastic modulus between the groups (Figure 8(f) and (g)), the control and 3% strain groups displayed the highest mean maximum strain before rupture (an increase of 87% initial length) whilst the 5% and 10% were lower (66% and 61%, respectively).

The engineered tendon-like tissues displayed viscoelastic biomechanical properties (Figure 8(h)–(k)). Percentage energy loss (Figure 8(i)) was calculated from the hysteresis runs: the 10% strain group had the lowest energy loss (47%) whilst the 5% strain group had the highest (60%), however no significance was seen. The rate of stress relaxation (Figure 8(k)) was however significantly higher in the 10% strain group as compared to the control (*p* = 0.036).

## Discussion

In the present study we used MSCs seeded within a fibrin scaffold to produce cell-engineered tendon-like tissues and applied cyclic tensile strain to promote tenogenic differentiation and maturation of the collagenous ECM. After tendon-like tissue formation by de novo cellular collagen synthesis and degradation of the fibrin scaffold, a 3D printed bioreactor chamber^25^ was used to apply cyclic strain during a further 21 days of culture. Gene expression of tenogenic markers and collagens was altered by the extent of cyclic strain, as was collagenous tissue composition, fibril diameter and fibre alignment. Biomechanical properties were altered with increased stress relaxation rate, a viscoelastic property, following 10% cyclic strain, but an increase in maximum stress following 3% cyclic strain.

Considering collagen gene expression, we observed increased expression of COL2A1, COL3A1 and COL11A1 with 5 h of 5% cyclic strain at 0.5 Hz for 21 days, but no significant increases with 10% strain or for COL1A1 expression. To our knowledge no prior study has systematically investigated the expression of the full repertoire of tendon-associated collagen in bioengineered tendon-like tissue, but in cyclically loaded live tendon fascicles (3%, 1 Hz cyclic plus 2% static strain) expression of COL6A3 and COL8A1 was increased after 24 h, with no significant differences were found for COL2, COL3, COL11 or COL12 as detected in our study. Herein COL2A1 expression was the lowest of all genes evaluated by qPCR, indicating that even non-dynamic tensile forces (control group) inhibit COL2A1 transcription, consistent with previously reported absent expression of COL2A1 from tenocytes given 24 h of cyclic 1% strain at 10 Hz or in tendon fascicles.^35,36^ For COL1 and COL3A1, a previous study in comparable fibrin hydrogels found that porcine MSCs subjected to 7 days of 10% cyclic tensile strain at 0.5 Hz increased COL1A1 but decreased COL3A1 expression.^12^ In other scaffolds alterations in COL1 and COL3A1 gene expression have been reported. Increased expression of COL1A1 and COL3A1 occurred with 2% and 4% strain at 0.1 and 1 Hz and with 6% strain at 0.1 Hz, for 2 h per day over 7 days^37^ and reportedly in a collagen sponge with 2.4% strain at 0.2 Hz for 8 h per day over 2 weeks.^38^ In crosslinked collagen scaffolds increased expression of COL3A1, but not COL1 has been reported,^39,40^ whilst in decellularized tendon or synthetic scaffolds, increased expression of COL1^41–43^ or both COL1 and COL3A1,^44–48^ with cyclic strain has been reported. However, in some situations expression of these collagens is unchanged.^35,49^ Hence differences in culture conditions and scaffold could account for differences in COL1 or COL3A1 expression.

COL12A1 was the most highly expressed mRNA of all the collagens but with significantly lower expression in the 5% and 10% strained groups, though no significant alterations in protein levels were detected by Western blotting. No type XIV was detected by Western blot suggesting that the engineered tendon-like tissues had matured beyond the developmental stage, or the type XIV antibody was less sensitive than that for type XII. qPCR results represent a snap-shot of gene expression at the end of the loading period, whereas protein abundance relates to accumulated collagen content following neotendon-like tissue formation and subsequent loading cycles. Alterations in gene expression may therefore not be maintained, or translated into protein, after 5 h cyclic loading and 19 h stasis.

For type I collagen, whilst there was an apparent proportional increase with strain (*R*^2^ = 0.9953, *p* < 0.01) differences between groups were not significantly different. An overall increase in collagen content with cyclic tensile strain has been reported for fibrin hydrogels,^12^ decellularized tendon^45^ and synthetic scaffolds^47^ and an increase in both type I and type III collagen by Western blotting was previously reported for rabbit tendon derived stem cells (TDSCs) in a synthetic scaffold with 4% cyclic strain, 0.5 Hz for 2 h per day over 14 days.^44^ In our system, type I collagen content may be established during neotendon-like tissue formation and remain largely unaltered with loading, whereas alterations in structural and biomechanical parameters may be independent of type I collagen content *per se*. The loading regimen was chosen following literature review (see Janvier et al.^25^), but it may be that more prolonged loading, or a more intermittent loading regimen would more profoundly alter collagenous protein content.

There was evidence of matrix mineralisation and increased alkaline phosphatase activity in the control and 10% strained groups (Supplemental Figure S2 and Figure 4), indicating that strain above or below physiologically acceptable levels has detrimental effects on the development of cell-engineered tendon-like tissue, consistent with previous work.^12^ Our objective in measuring alkaline phosphatase activity was to identify any evidence of osteogenic differentiation in the stem cells, as has previously been reported by application of overload strain in vitro^50^ and in vivo,^51^ and as a potential mechanism for calcific tendinopathies resulting from repetitive hyperextension.^52^ Collagen fibre alignment was greater in the mid-region than the anchor-point regions of the cell-engineered tendon-like tissues, as might be expected. Only the 10% strain group had greater collagen fibre alignment in the mid-region as compared to stasis. Cellular alignment was previously shown to increase with both 5% and 10% cyclic strain in fibrin hydrogels^12^ whilst ECM alignment was demonstrated to increase with cyclic strain in collagen hydrogels^37^ and in decellularized tissue scaffolds for pre-existing^46^ or newly synthesised ECM.^53^ It is unknown if fibre alignment is due to purely mechanical processes, or if cell-directed fibril tautening plays a role in orientation.

SHG was used for label-free visualisation of type I collagen. Combining GLCM texture analysis with PLS-DA showed separation of the samples by strain group and a different structure in the control group to the cyclically strained groups. These methods have the potential for non-invasive monitoring of bioengineered constructs in vitro or in vivo.

TEM was used to investigate how application of cyclic strain during culture affected collagen fibril maturation. Unlike in a previous study the addition of TGFβ3 was not required for the resorption of fibrin and deposition of collagen fibrils in stasis or with cyclical strain.^30^ 10% cyclic strain increased median collagen fibril diameter from 36.3 to 40.2 nm (a 19% increase). A previous study that applied slow stretch to double the length of similar fibrin-based tendon-like constructs over 4 days found an increase in mean fibril diameter from 34.4 to 38.4 nm (a 12% increase).^3^ Despite an increased fibril diameter with 10% cyclic strain, the observed collagen fibril diameters are still comparable to embryonic tendon.^13^ Application of cyclic strain during culture affected the intrinsic biomechanical properties of the resulting cell-engineered tendon-like tissues with 3% cyclic strain applied during culture increasing maximum stress (tensile strength) and 10% cyclic strain increasing the stress relaxation rate. We detected no differences in Young’s modulus for any cyclically loaded groups, consistent with a previous study which applied 2.4% cyclic strain to fibrin-based tendon-like constructs.^29^ However application of cyclic strain to collagen hydrogels at 2.4% strain or to poly-(ε-caprolactone) scaffolds at 3% strain did increase the final modulus.^38,54^ The increased maximum stress corresponds with a decreased collagen fibril diameter in the 3% strain group. A corresponding increase in maximum strain and/or modulus was not detected, presumably because stress/strain profiles are not linear throughout or identical for each replicate, reflecting inherent biological variability. Whilst fibril lengths were not measured as part of our experimental design, we hypothesise that fibrils length may have increased in the 3% strain group, alongside a decrease in fibril diameters, because increased tensile strength can occur with increased fibril length, particularly when a critical fibril length is surpassed.^5^ Redistribution of collagen molecules to the ends rather than the edges of the fibril with 3% strain could account for such alterations in fibril shape. Whilst the tensile strength approximately doubled, the final value (50–70 kPa) is less than 5% of that of native tendon.^5,55^ Hence a physiological increase in collagen fibril diameters, length and tensile strength in cell engineered tendon-like tissue may require a combination of slow stretching, cyclic strain or optimised concentrations of suitable combinations of growth factor cocktails.^27,56^

Increased stress relaxation can occur with loss of glycosaminoglycans (GAGs) or small leucine-rich repeat proteoglycans (SLRPs).^57–59^ GAGs may otherwise prevent fluid flow from the interfibrillar region into fibrils^60^ or out of the tissue^61^ hence attenuating the stress relaxation rate. Whilst the present study did not focus on GAGs or proteoglycans we noted a decrease in decorin gene expression in the cyclically strained groups, most substantially with 10% strain (Figure 1(c)). A proteomics analysis of strained tissues could in future be used to comprehensively assess alterations in protein composition. Loss of decorin also results in increased collagen fibril diameters,^62^ which we observed with 10% strain. Decorin null tendons have also been shown to have reduced maximum stress and modulus^62^ though we did not pick up significant differences for these parameters with 10% strain. Hence increased fibril diameter and stress relaxation with 10% strain could relate to reductions in decorin, other SLRPS or GAGs.

## Materials and methods

### Cell culture

hMSCs were derived from a bone marrow aspirate from a single normal (non-diabetic) adult (Lonza) and cultured at 37°C in normoxia and 5% CO_2_. The medium used throughout the investigation contained low glucose DMEM (Gibco, Life Technologies), 10% foetal bovine serum (FBS) (Gibco, Life Technologies), 2% antibiotic-antimycotic (Sigma-Aldrich), 1% nonessential amino acids (Sigma-Aldrich) and 1% L-glutamine (Sigma-Aldrich).

### Flow cytometry

The cell population was characterised by flow cytometry using an MSC verification kit (FMC020, R&D Systems) containing the International Society for Cell Therapy (ISCT) MSC positive markers CD73, CD90, CD105 (validation threshold at >95% population) and a negative marker cocktail containing CD11b, CD34, CD45, CD79A and HLA-DR (validated at ~2% of the cell population). The kit was used as per manufacturer’s protocol on the cell population at passage 1. Antibody compensation was performed using the BD™ CompBeads (552843, BD Bioscience) and analysed by the BD FACS Canto II flow cytometer using FlowJo software.

### Tri-linage differentiation assays

hMSCs were expanded on tissue culture plastic up to passage 2 and disassociated from the tissue culture plastic by trypsin. Cells were re-plated in triplicate at seeding densities based on the required differentiation for each assay: for both osteogenic and adipogenic differentiation the cells were seeded in monolayer in the wells of a six-well plate. For osteogenic differentiation, cells were seeded at 3000 cells/cm^2^ and cultured in osteogenic media containing 100 nM dexamethasone, 10 mM β-glycerophosphate and 50 μM L-ascorbic acid (all Sigma Aldrich UK).^63^ For adipogenic differentiation the cells were seeded at 10,000 cells/cm^2^ and cultured in adipogenic media containing 1 μM dexamethasone, 100 μM indomethacin, 10 μg/mL insulin and 500 μM 3-Isobutyl-1-methylxanthine (IBMX) (all Sigma Aldrich UK).^64^ For chondrogenic differentiation the cells were pelleted from 500,000 cells in 500 μL of basic media in a 1.5 mL screw cap centrifuge tube, centrifuged at 240*g* and media replaced with chondrogenic media containing 10 ng/mL transforming growth factor β3 (TGF-β3) (Peprotech), 100 nM dexamethasone, 1% Insulin-Transferred-Sodium (ITS), 25 μg/mL L-ascorbic acid (all Sigma Aldrich UK).^65^ Controls in the standard non-differentiation (proliferation) medium were cultured identically to differentiating groups. Both osteogenic and adipogenic groups had the media changed twice a week whilst the chondrogenic group had the media changed every 48 h. To assess differentiation the groups were stained for calcium with Alizarin Red (osteogenic differentiation), Oil-Red-O (adipogenic differentiation), or Alcian blue (chondrogenic differentiation).

### Cell-engineered tendon-like tissue fabrication

The generation of the cell-engineered tendon-like tissue using a fibrin-based hydrogel was performed as previously described.^25^ Briefly, 3D printed ‘tendon attachment frames’ were sterilised in 70% ethanol and washed in sterile PBS before being fixed into position within a six-well plate by dispensing 2.5 mL sterile 4% agarose around the outside of the frame. MSCs were dissociated from the tissue culture plastic at passage 3 using trypsin and seeded at 1.25 × 10^6^ cells/mL in fibrin to create individual cell-engineered tendon-like tissues within the frame from 75 μL (20 mg/mL) fibrinogen, 25 μL (200 Units) thrombin (both Sigma-Aldrich) and 230 μL media containing the cell suspension. The hydrogels were cultured in the six-well plate for 14 days with media changes every 48 h. 1 mg/mL 6-aminocaproic acid (Sigma-Aldrich) was added to the media to inhibit fibrin degradation during the contraction phase.^66^ After 14 days, the ‘tendon attachment frames’ were moved from the six-well plate and placed into separated wells within the bioreactor chamber. Once in the bioreactor chamber, the frame-connecting spars were broken using sterile scissors. The cell-engineered tendon-like tissues were cultured in 3.5 mL of media with 800 μM of freshly prepared L-ascorbic acid (Sigma-Aldrich) and media changes every 48 h.

### Mechanical stimulation and experimental design

Uniaxial cyclic tensile strain was applied to the cell-engineered tendon-like tissues using a universal 3D printed bioreactor chamber^25^ mounted onto an EBERS TC3. The bioreactor was housed within a non-humidified incubator set at 37°C in normoxia and 5% CO_2_. The cell-engineered tendon-like tissues were loaded at either 3%, 5% or 10% strain (based on the initial 8 mm length of the tissue-engineered tendon) for 5 h per day at 0.5 Hz for 5 consecutive days per week over 21 days. The loading regimen was chosen based on our previous research^25^: there is little consistency in comparable studies, 0.5 Hz was the upper frequency limit for the bioreactor, 5 h loading per day mimics diurnal activity with two rest days, and 3%, 5% and 10% strain were chosen to mimic low, physiological and high tendon strains.^2^ Control samples were placed in a bioreactor chamber with a fixed tensile arm, designed to transfer no cyclic strain and cultured in the same incubator as bioreactor loaded samples.

A total of six experimental runs were performed, using three separate bioreactor chambers. Each chamber was used for two different loading conditions to account for any hardware variation between bioreactor chambers. Each run was staggered to optimise experimental run time (20 weeks), with each run made up of a 2 weeks contraction and a 3 weeks loading phase. The three loading bioreactor chambers were rotated for each run, that is chamber 1 was used for 3% during run 1, 5% during run 2 and 10% during run 3 and then repeated for runs 4–6. Samples for experimental repeats were selected from across the six bioreactor wells by random determination using an online tool (www.random.org/sequences/) and varied across each run. In run #1, #2 and #3 two samples from each loading condition were taken for western blot analysis, and two samples for qPCR analysis, with two spare (*n* = 6 total). In run #4, #5 and #6 one sample was taken for histology, one sample for collagen probe imaging, one sample for mechanical testing, and one sample for TEM with two spare samples (again *n* = 6 total).

### RNA isolation and Real-time Reverse Transcription polymerase Chain Reaction

A total of six samples from each group were removed from the bioreactor chamber at the end of the culture period and transferred to RNase- and DNase-free 2 mL centrifuge tubes. 300 μL of TRIzol reagent (ThermoFisher) was added to each centrifuge tube and samples were frozen at −80°C before analysis. Thawed samples were homogenised using the TissueRuptor II (Qiagen) before being drawn up into a 1 mL syringe and passed through a 23 gauge needle into a fresh 1.5 mL centrifuge tube. RNA was isolated from each sample using the phenol chloroform phase separation method and the RNA pellet suspended in RNase-free water. RNA was extracted from monolayer of hMSCs by the phenol chloroform phase separation method. Initially cells were disassociated from the tissue culture plastic by trypsinisation at passage 3, pelleted and re-suspended in TRIzol at 1,500,000 cells/mL. Extracted RNA was suspended in RNA-free water. The quality and quantity of RNA was measured using the NanoDrop 2000 spectrophotometer (Thermo Scientific). cDNA was synthesised using an iScript kit (BioRad). The qPCR reaction was performed using a Stratagene MX3005P instrument with each 10 μL reaction containing 10 ng cDNA, 2.8 μL distilled water, 5 μL SYBR Green JumpStart *Taq* ReadyMix (Sigma Aldrich UK), 5 μM Forward Primer, 5 μM Reverse Primer and 1x reference dye (ROX) (Sigma Aldrich UK). Primers used and gene abbreviations are listed in Table 1. For tendon marker genes (DCN, SCXA and TNMD) the experimental samples (control, 3%, 5% and 10%) from three separate experimental runs (*n* = 3) plus monolayer hMSC samples (*n* = 3) were used. For collagen genes (COL1A1, COL3A1, COL5A1, COL11A1, COL12A1, COL14A1 and COL2A1) *n* = 3 experimental samples were used from three separate runs. The amplification profile used for qPCR was 95°C for 5 min then 40 cycles at 95°C for 10 s, 55°C for 10 s and 68°C for 30 s. The most suitable housekeeper gene was confirmed using GeNorm by comparing stability of expression for the following genes: GAPDH, GUSB, HPRT1, TBP and YWHAZ. YWHAZ was confirmed as the most stable gene and was used as the reference gene for comparison. Analysis was carried out using the efficiency corrected ∆CT method as previously described.^67,68^

**Table 1.** Primer sequences.

Gene	Forward	Reverse	Origin
COL1A1	CGGCTCCTGCTCCTCTTAG	CACACGTCTCGGTCATGGTA	Naranda et al.^69^
COL2A1	CCAGATTGAGAGCATCCGC	CCAGTAGTCTCCACTCTTCCAC	Naranda et al.^69^
COL3A1	GGGTGAGAAAGGTGAAGGAG	CATTACTACCAGGAGGACCAG	Designed
COL5A1	NM_000093, QIAGEN	QIAGEN	
COL11A1	AATGGAGCTGATGGACCACA	TCCTTTGGGACCGCCTAC	Karaglani et al.^70^
COL12A1	TTTAGTTAGCACAGCGGGCA	CGCTCGAAATACACAGCAGC	Designed
COL14A1	NM_021110, QIAGEN	QIAGEN	
DCN	NM_133503, QIAGEN	QIAGEN	
SCXA	NM_001080514, QIAGEN	QIAGEN	
TNMD	NM_022144, QIAGEN	QIAGEN	
YWHAZ	CCGTTACTTGGCTGAGGTTG	TGCTTGTTGTGACTGATCGAC	Lemma et al.^71^

### Protein analysis

A total of six samples from each group were removed from the bioreactor chamber at the end of the culture period, frozen at −80°C, thawed and ground using a motorised pestle (Sigma-Aldrich). Soluble protein was extracted in RIPA buffer (Sigma-Aldrich), and insoluble material was digested in 25 μg/mL pepsin (Sigma-Aldrich), 0.1 M acetic acid (Sigma-Aldrich) and 200 μg/mL EDTA (Sigma-Aldrich). Soluble and digested protein were combined and total protein concentration measured using the BCA assay. 15 μg of extracted protein was electrophoresed using the Criterion Vertical Electrophoresis system (Bio-Rad) with NUPAGE 3%–8% Tris-Acetate midi protein gels (ThermoFisher). Electrophoresed proteins were transferred to nitrocellulose membrane and total protein was stained for using REVERT Total Protein Stain (Li-cor) and imaged with the Odyssey CLx imaging system (Li-cor). The membrane was washed with Tris-buffered saline with Tween 20 (TBS-T) buffer and then blocked for 1 h at room temperature with 5% reconstituted dehydrated milk in TBS-T (blocking buffer). The membrane was then incubated overnight at 4°C with either anti-collagen α1(I) (1:1000) (ab138492, Abcam), anti-collagen α1(II) (1:500) (sc-52658, Santa Cruz), anti-collagen α1(III) (1:1000) (ab7778, Abcam), anti-collagen α1(V) (1:500) (ab112551, Abcam), anti-collagen α1(XI) (1:500) (ab64883, Abcam), anti-collagen α1(XII) (1:500) (ab121304, Abcam) or anti-collagen α1(XIV) (1:500) (ab101464, Abcam) primary antibodies in blocking buffer. The primary antibody was removed and the membrane washed three times with TBS-T before incubating at room temperature for 1 h in the anti-rabbit secondary antibody (1:5000 in blocking buffer) (926-32213, Li-cor) (for collagen α1(I), α1(III) and α1(XII)) or the anti-mouse secondary antibody (1:5000 in blocking buffer) (926-3312, Li-cor) (for collagen α1(II), α1(V), α1(XI) or α1(XIV)). The membrane was washed a further three times with TBS-T. Antibody fluorescence was detected by imaging using the Odyssey CLx imaging system. Densitometry was performed using EmpiriaStudio (Li-cor) with expression normalised to total protein.

### Histology

Two cell-engineered tendon-like tissues from each group were removed from the bioreactor chamber, washed three times in PBS and fixed in 10% neutral buffered formalin (NBF) for 30 min at room temperature. Fixed samples were washed three times in PBS and stored in 0.01% sodium azide in PBS at 4°C, then mounted in paraffin wax and sectioned using a Leica RM2245 microtome, generating 6 μm slices. The sections were stained for Haematoxylin and Eosin (H&E), Picrosirius red, Alcian blue and Alizarin red and imaged using a Nikon Eclipse Ci with a ×10 objective.

### Alkaline phosphatase

Culture medium was collected at weekly time points throughout the experimental run (days 0, 7, 14 and 21). From the collected culture medium 50 μL was dispensed into a 96 well plate, along with 100 μL of p-nitrophenyl phosphate, a phosphatase substrate that turns yellow (λmax = 405 nm) when dephosphorylated by alkaline phosphatase after 10 min of incubation at room temperature. Standard curves were generated with known concentration of bovine mucosal alkaline phosphatase (Sigma-Aldrich UK).

### CNA35 collagen-probe

Following the cyclic loading period three samples from each group were fixed in 10% NBF (Sigma Aldrich UK) for 30 min within the 3D printed bioreactor chamber. The samples were then sectioned using a vibrotome (Leica Microsystems) as previously described.^72^ Sections were stained using the CNA35 collagen-probe as described,^73^ with the addition of 1:1000 DAPI (ThermoFisher) for 20 min during the final wash stage. Once stained, samples were stored overnight in a final change of TBS at 4°C. Sections were imaged using a Confocal LSM800 microscope (Zeiss) and image analysis performed using FIJI (ImageJ) with the plugin OrientationJ (BIG).^74^ Images were taken in two distinct regions; the mid region and the anchor point region.

### Second harmonic generation

Optical experiments used a two-photon microscope described in our previous efforts.^75,76^ Second harmonic generation (SHG) images of collagen and cells stained with AF488 C_5_ Maleimide (ThermoFisher) were produced using a linearly polarised Ti:Sapphire excitation laser. SHG allows for label-free, non-destructive imaging of collagen type I, with intensity dependent on collagen quantity, directionality and organisation. Analysed images were 5122 × 512 pixels and an average of 300 frames that received a 0.5 sigma Gaussian blur.

The grey-level fluctuations of the collagen appeared different among the different stress strengths. To analyse these textural differences, pixel intensities were binned into 20 grey levels and were evaluated using the grey-level co-occurrence matrix (GLCM) contrast, correlation, energy and homogeneity terms.^77^ Contrast describes the intensity contrast across the image, correlation describes the linear dependency of pixel pairs, energy describes the orderliness of the image, and homogeneity describes the distribution of the grey levels in the image.^77^ Although images were taken in the same orientation, any minor variations in fibre direction were corrected using a method described by Hu et al.^78^ to so that the maximum correlation values were along the *y*-axis; thus, the dominant angle of collagen fibres within the image was at 90°. Each measurement was determined between pixel pairs that are at 0°, 45°, 90° and 135° from each other and of 0 to 3.3 µm (~50 pixels) away. Each stress strength had two replicates and each replicate had four images analysed.

GLCM analysis provides a rich data set comprising hundreds of data points for each image. To reduce the dimensions and investigate key features that differed in collagen organisation with the addition of mechanical strain, we performed partial least squares discriminant analysis (PLS-DA). The method allows for the separation of the two groups by assigning weighted coefficients to the response variables using a linear model, with large absolute values of the coefficient being assigned to variables that have great influence on separating the groups. Once computed, we evaluated the error using leave-one-out cross-validation.

### Transmission Electron Microscopy (TEM)

At the end of the culture period three samples were removed from each group which underwent primary fixation in 2.5% glutaraldehyde (Taab) in 0.1 M sodium cacodylate buffer (Taab) for 4 h at 4°C. The samples underwent secondary fixation with contrast stain in 1% aqueous osmium tetroxide (Taab) for 1 h before ‘en bloc’ staining using 2% uranyl acetate (Taab) in 0.69% maleic acid (Taab) for 1 h. Samples were dehydrated through 50%, 70%, 90% and 100% ethanol and finally 100% acetone. The samples were then embedded in ‘Taab’ epoxy resin (Taab) through changing ratios of resin to acetone, initially 1:3 for 1 h, then 2:2 for 1 h and 3:1 for 1 h before two changes of 100% epoxy resin for 1 h each and overnight polymerisation at 60°C. Samples were cut to ultrathin sections (60–90 nm) using a ‘Diatome’ diamond knife (Agar Scientific) on a Reichert-Jung Ultracut ultramicrotome (Leica Microsystems). Sections were mounted onto a 200 mesh copper grid (Agar Scientific), counter stained with saturated uranyl acetate in 50% methanol followed by Reynolds Lead Citrate stain (Taab) and imaged using a Phillips EM208S Transmission Electron Microscope at 80 kV. Analysis of the minimum diameter of elliptical collagen fibril profiles, as a proxy for collagen fibril diameter, in images of transverse sections was performed using FIJI (ImageJ) as previously described.^79^

### Mechanical testing

**Figure A1.**
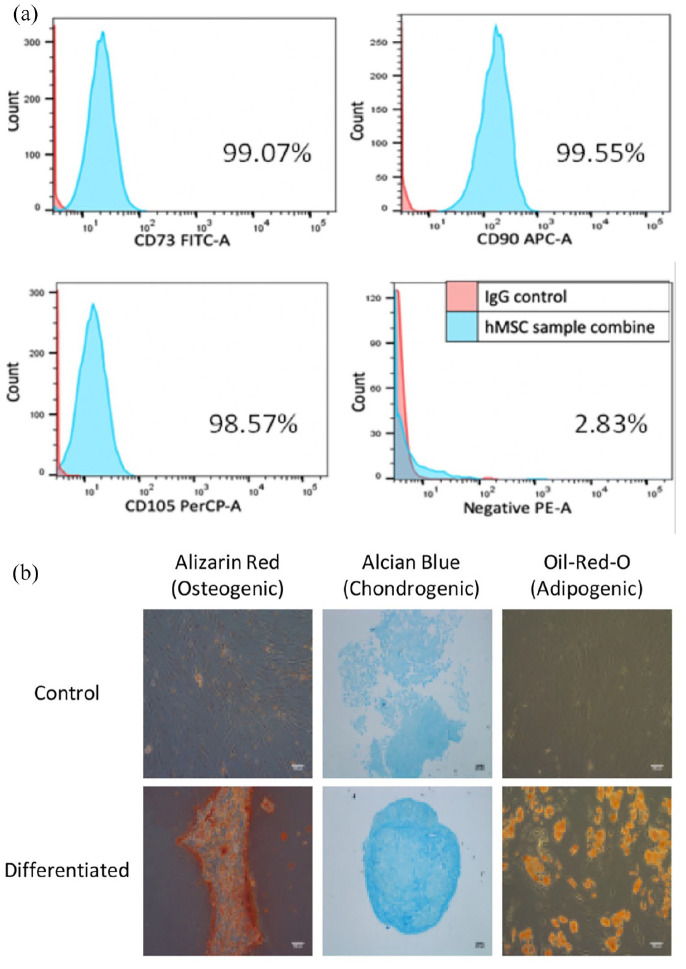
Graphical schematic of tensile testing loading regime. During the first 120 seconds the samples underwent pre-conditioning, which involved 30 s of extension by 20% followed by relaxation and resting for 90 s. The sample then followed this profile for the next 360 s for hysteresis analysis. Following hysteresis, the samples were tested for stress relaxation by being stretched by 20% and held in position for 60 s before relaxation and rest for 90 s. Samples were finally loaded until failure.

Mechanical testing was performed on three samples from each stimulation group at the end of the culture period. Before removal from the bioreactor chamber, each sample was imaged using a HD USB camera (MicroDirect, Celstron) and the average diameter of the tissue engineered tendon was recorded to calculate the cross sectional area of the sample.^3,26,80^ The known width of each well (15 mm) was used as measurement reference. The engineered tendon samples were placed onto paper frames to act as support structures during transfer from bioreactor chamber to the tensile testing system; a Univert (CellScale) with a 1 N load cell. Once placed within the grips the paper frame was broken and the tissue engineered tendon was held freely between the grips. The samples were tested for hysteresis, stress relaxation and stiffness with pre-conditioning performed during the initial cycles of hysteresis testing (Figure A1). Young’s modulus was calculated by finding the stress and strain from the linear region of the stress-strain graph for each sample that was mechanically tested. Following tensile testing the mechanical properties were calculated using the following equations:

$$Stress:\sigma =\frac{F}{A}$$

Where F is the recorded force and A is the cross sectional area (before loading)

$$Strain:\varepsilon =\frac{\Delta l}{l}$$

Where Δl is the change in length and l is the initial length (8 mm)

Hysteresis(energydissipitated)=Areabetweencurves

$$\begin{array}{l} Hysteresis=Area\;below\;loading\;curve-\\ Area\;below\;unloading\;curve \end{array}$$

$$Percentage\;Energy\;loss=\frac{Hysteresis}{Area\;below\;loading\;curve}\times 100$$

$$Rate\;of\;change\;of\;Stress=\frac{\sigma_{0}-\sigma_{60}}{T}$$

Where σ_0_ is the initial stress (at 0 s), σ_60_ is the final stress (at 60 s) and T is the relaxation time (60 s during this study).

$$Young’s\;Modulus:E=\frac{\sigma }{\varepsilon }$$

### Statistical analysis

One-way analysis of variance (ANOVA) with Tukey’s post hoc test and multiple comparison tests for gene analysis, western blot analysis, collagen probe analysis and mechanical testing was carried out using with GraphPad Prism 8. Two-way ANOVA with Tukey’s post hoc test and multiple comparisons was used for the Alkaline Phosphatase analysis. Where residuals did not meet assumptions of normality (Shapiro-Wilk test) or equal variance (Brown-Forsythe test) data were transformed before analysis. If a suitable transformation was not identified data was analysed using the Kruskal-Wallis test. Statistical analysis of collagen fibril diameters using TEM was undertaken in R (R, Version 4.0.3).^81^ The lmer function from the package lme4 was used to fit linear mixed models to assess differences in measured outcome variables between different loading conditions.^82^ In all cases, the measured outcome was specified as the response variable, the loading condition as the fixed effect and the run of the experiment as the random effect. For TEM analysis the means of the minimum diameters were determined for each image and different images accounted for with the specification of run as a random effect. Estimated marginal means and Tukey adjusted p values were extracted using the emmeans package.^83^

## Conclusions

In this investigation we focused on quantifying the total repertoire of detectable collagen isoforms, which we aim to further develop as a characterisation ‘fingerprinting’ biomarker method to link tissue engineered tendon biochemistry to functional outcomes. This approach shows that de novo tenogenesis by adult hMSCs appears to occur via a partial recapitulation of developmental processes (which occurs through a series of stages that are associated with transitory and fibril-modifying collagen isoforms) rather than simple extracellular matrix remodelling by tissue-resident stem cells. The multimodal analysis we conducted involved probing as many isoforms as were detectable at both transcript and mature protein level, and matching these data to functional outcome measures (mechanical testing). We correlated the collagen isoform composition of these de novo tissues with imaging data at different scales, our objectives being to provide evidence to support a mechanistic link between collagen isoforms and ultrastructural differences in fibril architecture.

Most of the parameters we tested had a non-linear response to increasing cyclic strain during culture, although fibre alignment (which increased proportionally with increasing cyclic strain) was a notable exception. The extent of cyclic strain influenced collagen isoform expression, fibril diameter and fibre alignment as well as the mechanical properties of the cell-engineered tendon-like tissues (3% cyclic strain increased maximum stress two-fold). The 5% cyclic strain group showed increased expression of the tenogenic markers scleraxis and tenomodulin, as well as COL2A1, COL3A1 and COL11A1 genes but significantly lower expression of COL12A1. COL12A1 expression was also decreased in 10% strain as was type II collagen protein content. The 10% strain group had increased expression of scleraxis, increased collagen fibre alignment in the mid-region and increased collagen fibril diameter, as well as a higher stress relaxation, indicative of an improved ability to alter its internal structure in response to external stress.

The above objectives were made possible by our customised bioreactor chamber, which was designed by the authors and 3D printed^25^. The design allowed for six experimental replicates (per chamber) and efficient handling, since the entire chamber could be unbolted and replaced within minutes without disturbing the individual tendons. This design strategy enabled a complex multimodal analysis within a reasonable time frame, with enough experimental power to yield statistically significant results for each analysis type. The chamber design we have previously published allows direct reproduction of our experimental method using any of three common bioreactor base platforms (EBERS-TC3, CellScale Mechanoculture T6 and the TA ElectroForce series)^25^. In our future work we will further explore the utility of collagen isoform composition as a biomarker for functional maturation of hMSC-derived tissues.

## Supplemental Material

sj-docx-1-tej-10.1177_20417314221130486 – Supplemental material for Multimodal analysis of the differential effects of cyclic strain on collagen isoform composition, fibril architecture and biomechanics of tissue engineered tendon Click here for additional data file. Supplemental material, sj-docx-1-tej-10.1177_20417314221130486 for Multimodal analysis of the differential effects of cyclic strain on collagen isoform composition, fibril architecture and biomechanics of tissue engineered tendon by Adam J Janvier, Emily G Pendleton, Luke J Mortensen, Daniel C Green, James R Henstock and Elizabeth G Canty-Laird in Journal of Tissue Engineering
