# Supplementary material for: Multimodal analysis of the differential effects of cyclic strain on collagen isoform composition, fibril architecture and biomechanics of tissue engineered tendon
Source: J Tissue Eng. 2022 Oct 31;13:20417314221130486. doi: 10.1177/20417314221130486 (PMC9629721; doi:10.1177/20417314221130486)
Supplement: sj-docx-1-tej-10.1177_20417314221130486 – Supplemental material for Multimodal analysis of the differential effects of cyclic strain on collagen isoform composition, fibril architecture and biomechanics of tissue engineered tendon [file sj-docx-1-tej-10.1177_20417314221130486.docx]

**Supplementary Figures**


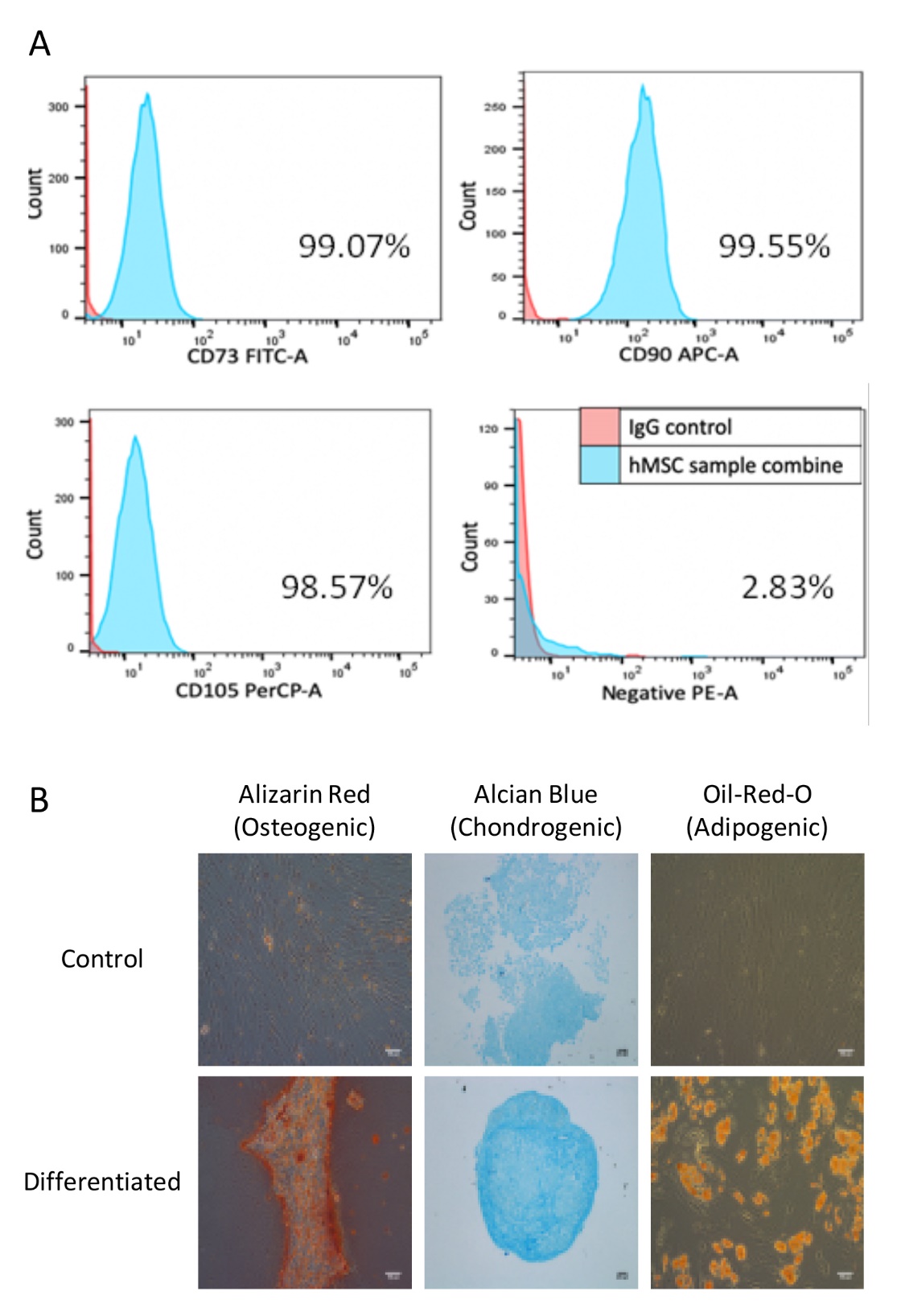


**Figure S1. Characterisation of human MSCs.** A) hMSCs were characterised using fluorescence activated flow cytometry in accordance with the ISCT panel of surface markers showing positive expression for CD73 (99.07%), CD90 (99.55%) and CD105 (98.57%) with low expression of the negative marker cocktail: CD14, CD19, CD34, CD45 and HLA-DR (2.83%). B) hMSCs were shown to be capable of trilineage differentiation into osteoblasts, chondrocytes and adipocytes following 21 days of culture in appropriate cell culture medium. Osteogenic differentiation measured using alizarin red staining, chondrogenic differentiation with Alcian blue staining and adipogenic differentiation with Oil-Red-O staining. n=3.


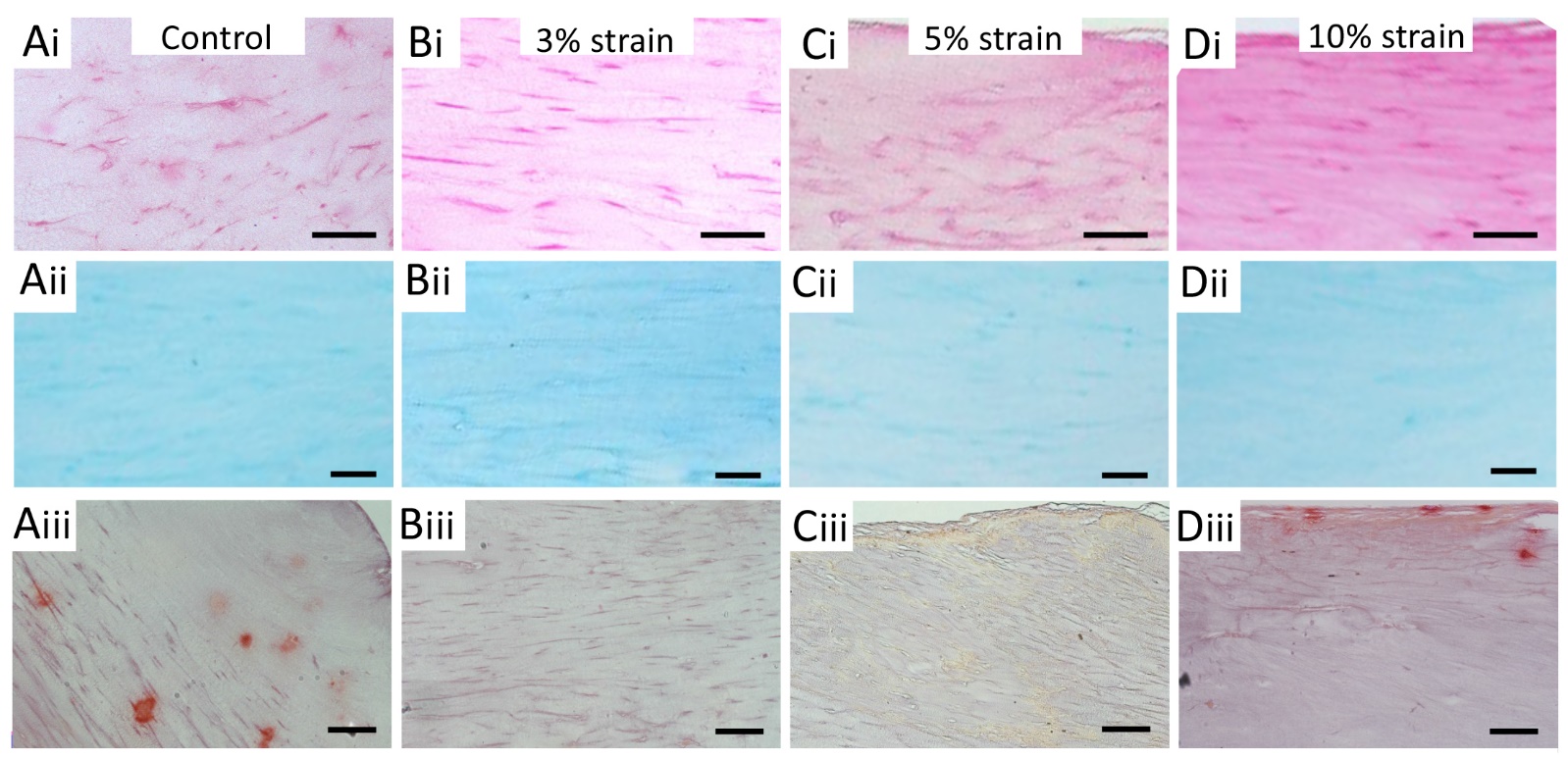


**Figure S2. Histological analysis of cell-engineered tendon-like tissues.** Cell-engineered tendon-like tissues were cultured with 21 days of intermittent cyclic tensile strain and then fixed and sectioned. Serial sections were stained to visualize cell bodies with H&E (Ai-Di), and ECM components characteristic of differentiation. Alcian blue staining (Aii-Dii) was used to visualize glycosaminoglycans, whilst Alizarin red (Aiii-Diii) was used to detect any calcification in the tissues. Images are representative of n=2 experimental repeats. Scale bars are 100µm.


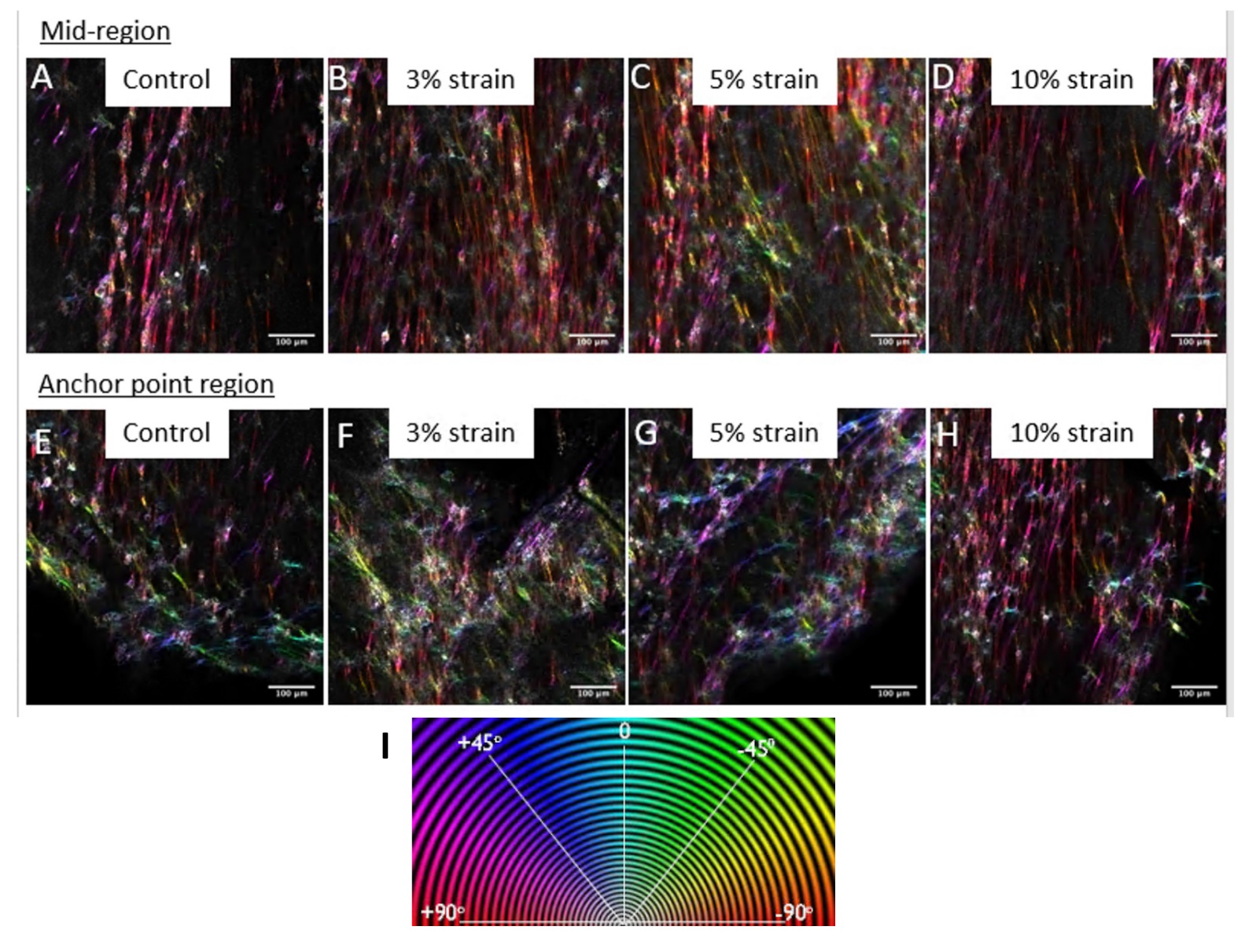


**Figure S3. Visualised orientations of collagen fibres in cell-engineered tendon-like tissues.** Collagen was visualised using a CNA35 fluorescent probe as shown in Fig. 5, and stained fibre orientations visualised using the Fiji plug-in OrientationJ. Images show anchor point (AR) region (A-D) and the middle (MR) region (E-H) of tendon-like tissues cultured without dynamic strain (A, I; control) or with 3% cyclic strain (B, J), 5% cyclic strain (C, K) or 10% cyclic strain (D, L).(I) shows the colour scale applied by the OrientationJ software. Images are representative of n=3 experimental repeats and scale bars represent 100µm.
